# Screening and Metabolomic Analysis of Lactic Acid Bacteria-Antagonizing *Pseudomonas aeruginosa*

**DOI:** 10.3390/foods12142799

**Published:** 2023-07-23

**Authors:** Jianzhou Li, Xiaohua Chen, Ziyan Xie, Lin Liang, Anping Li, Chao Zhao, Yuxi Wen, Zaixiang Lou

**Affiliations:** 1 College of Food Science and Engineering, Central South University of Forestry and Technology, Changsha 410004, China; joux123@gmail.com; 2 Hunan Key Laboratory for Conservation and Utilization of Biological Resources in the Nanyue Mountainous Region, College of Life Sciences, Hengyang Normal University, Hengyang 421008, China; chxh0217@163.com (X.C.); yolanda230719@163.com (Z.X.); 3 Department of Life Sciences, Nanyue College of Hengyang Normal University, Hengyang 421008, China; 15575440140@163.com; 4 College of Marine Sciences, Fujian Agriculture and Forestry University, Fuzhou 350002, China; zhchao@live.cn (C.Z.); yuxi.wen@outlook.com (Y.W.); 5 Nutrition and Bromatology Group, Department of Analytical and Food Chemistry, Faculty of Sciences, Universidade de Vigo, 32004 Ourense, Spain; 6 State Key Laboratory of Food Science and Technology, School of Food Science and Technology, Jiangnan University, Wuxi 214122, China

**Keywords:** lactic acid bacteria, *Pseudomonas aeruginosa*, metabolomic analysis

## Abstract

*Pseudomonas aeruginosa* is a conditional Gram-negative pathogen that produces extracellular virulence factors that can lead to bloodstream invasion, severely harm tissues, and disseminate bacteria, ultimately leading to various diseases. In this study, lactic acid bacteria (LAB) with strong antagonistic ability against *P. aeruginosa* were screened, and the regulatory mechanism of LAB against *P. aeruginosa* was evaluated. The results showed that the three selected LAB strains had strong inhibition ability on the growth, biofilm formation, and pyocyanin expression of *P. aeruginosa* and a promoting effect on the expression of autoinducer-2. Among them, *Lactipantibacillus plantarum (Lp. plantarum)* LPyang is capable of affecting the metabolic processes of *P. aeruginosa* by influencing metabolic substances, such as LysoPC, oxidized glutathione, betaine, etc. These results indicate that LPyang reduces the infectivity of *P. aeruginosa* through inhibition of its growth, biofilm formation, pyocyanin expression, and regulation of its metabolome. This study provides new insights into the antagonistic activity of *Lp. plantarum* LPyang against *P. aeruginosa*.

## 1. Introduction

Foodborne pathogens are an important contributor to food safety problems and can directly or indirectly contaminate food and water sources, thereby affecting human health. With its high regulatory capabilities, *Pseudomonas aeruginosa* (*P. aeruginosa*) is a ubiquitous Gram-negative foodborne opportunistic pathogen that can survive in different environments, being present in the soil, water, and different animal hosts [1]. *P. aeruginosa* is one of the highly infectious pathogens in the food industry and can exist in water, dairy products, meat, and plant-derived foods, and can even affect the physicochemical properties of food, such as nitrite content, resulting in food spoilage or poisoning [2]. Also, it can adhere to various surfaces of equipment used in food processing, where it can form biofilms, threatening food safety and quality and possibly causing foodborne illness [3]. Pyocyanin production by *P. aeruginosa* has a significant impact on food safety. Pyocyanin, an important virulence factor of *P. aeruginosa*, is also the terminal response signal molecule of *P. aeruginosa*’s quorum-sensing (QS) system. The current findings showed that the QS system of *P. aeruginosa* had a certain correlation with the secretion of virulence factors such as pyocyanin [4]. The biofilm formed by *P. aeruginosa* brings serious health risks to the food-processing environment and is also a key factor that endangers food safety. Burnett et al. [5] found that bacteria adhering to the surface of apples formed a biofilm that invaded the fruit tissue and caused spoilage. More researchers have attempted to reduce the virulence factor production of *P. aeruginosa*, inhibit its formation of biofilms, and inhibit its toxic effects in foodstuffs by interfering with the QS system of *P. aeruginosa* [6]. Currently used antimicrobials may cause contamination of meat during slaughter or processing of livestock, poultry, and aquatic products, as well as on fruits, vegetables, or other products irrigated with contaminated water, and transmitted to humans through contact after ingestion of these contaminated foods, resulting in damage to human health [7]. Furthermore, overused and/or misused antibiotics have dramatically accelerated the acquisition of antimicrobial resistance of *P. aeruginosa*, which greatly accelerates its development into antibiotic-resistant strains [8]. Therefore, finding safe and natural alternatives to chemical antibiotics and reducing the advancement of antimicrobial resistance have become increasingly important strategies for treating *P. aeruginosa* infection.

Lactic acid bacteria (LAB), a large heterogeneous group of Gram-positive, non-spore-forming bacteria, have numerous advantages. It is an important class of beneficial microorganisms that are recognized as safe food-grade microorganisms and can be used in place of antibiotics in the production of food. LAB, widely and traditionally used in fermented foods, are mainly used to improve the functional characteristics and added value of food and increase the nutritional value of food. LAB can also inhibit the growth of pathogenic bacteria and spoilage bacteria in food, extending the shelf life of food [9]. LAB can reduce serum cholesterol and stimulate the production of some antibacterial substances by suppressing the growth of spoilage bacteria in the gastrointestinal system [9]. LAB have the advantages of safety, non-toxic side effects, and high efficiency. The metabolites that LAB produce, such as hydrogen peroxide, acids, and bacteriocin, can effectively stop the growth of some harmful microbes [10]. LAB have a certain antagonistic effect on *P. aeruginosa*. *Pediococcus pentosaceus* isolated from fish and shrimp intestines had different inhibitory effects on *Vibrio vulnificus*, *P. aeruginosa*, and *Escherichia coli* [11]. *Latilactobacillus sakei* LY1-6 screened from the ocean perch intestine had strong inhibitory activity against *Pseudomonas fluorescens,* and its antibacterial substance was determined to be bacteriocins [12]. Srikanjana discovered that the antibacterial effect of LAB was through the destruction of the cell membrane of other pathogenic bacteria, resulting in the shrinkage or cracking of pathogenic cells [13]. 

Hence, in this study, LAB with activity against *P. aeruginosa* were screened, and the function of the LAB was explored by metabolomic analysis to lay a foundation for studying the mechanism of LAB against *P. aeruginosa*.

## 2. Materials and Methods

### 2.1. Bacterial Strains and Growth Media

*P. aeruginosa* PAO1, 17 strains of LAB (H1–17, Table 1), *Vibrio harveyi* BB170, and *V. harveyi* BB152 were obtained from Hunan Key Laboratory for Conservation and Utilization of Biological Resources in the Nanyue Mountainous Region, Hengyang Normal University. The culture media (de Man, Rogosa, and Sharpe medium (MRS medium)), autoinducer bioassay medium (AB medium), Luria–Bertani medium (LB medium), and marine culture medium were purchased from Hopebio Co., Ltd. (Qingdao, China). The strains were activated three times and grown in a medium for 18 h, and optical density (OD) was adjusted at 600 nm.

### 2.2. Preparation of Supernatants for Test Strains

*V. harveyi* BB170 was activated by three subcultures in AB medium at 2% inoculum size with shaking at 120 rpm at 30 °C. After that, the culture continued to cultivate for 12 h. LAB strains and *P. aeruginosa* were cultured in three subcultures at 37 °C for 18 h in a MRS and LB medium, respectively. Additionally, the culture supernatant was obtained by centrifugation (6000 rpm, 10 min) and filtered with a 0.22 μm sterile membrane. 

### 2.3. Preparation of Co-Culture Supernatant of LAB and P. aeruginosa

*P. aeruginosa* was activated three times and grown in LB broth with shaking at 150 rpm at 37 °C for 12 h. *P. aeruginosa* (adjusted for 10^6^ cfu/mL) at logarithmic phase was inoculated in LB broth supplemented with LAB strain supernatants, MRS medium, and LB medium (ratio of 5%) and incubated with shaking at 150 rpm for 24 h at 37 °C. Centrifugation (6000 rpm, 20 min) was used to collect the culture supernatants, which were then filtered with a 0.22 μm sterile membrane.

### 2.4. Assay to Measure LAB Strains’ Inhibitory Ability 

The inhibitory ability of different LAB strains was evaluated following a previously reported method with some modifications [14]. *P. aeruginosa* was cultured on fresh LB agar plates (10^8^ cfu per plate), and a 6 mm disc was placed in it. Then, 150 μL of fresh *Lactobacillus* culture was added to the discs. The diameter of the inhibitory zone surrounding the discs was determined after the cultures were grown from 18 to 24 h at 37 °C. The negative control utilized MRS media (pH 4.0).

### 2.5. Biofilm Inhibition Assay

The biofilm inhibition rate was tested using the method of Rana et al. [8] with a few modifications. *P. aeruginosa* was activated three times and grown in LB broth with shaking at 150 rpm at 37 °C for 12 h. An optical density (OD) of 0.05 was adjusted by LB broth at 600 nm. Equal volumes of *P. aeruginosa* cell suspension (0.05 OD) and the LAB’s cell-free supernatant (ratio of 1%) were combined in each disc of the 96-disc microplate and incubated for 24 h at 37 °C. The supernatant was removed after the incubation period, and distilled water was used to wash the microplate discs. The biofilm was solubilized with 95% ethanol and stained with 0.1% crystal violet (CV). Absorbance was measured at 570 nm using a microplate reader (FLUOstar Omega, BMG Labtech, Ortenberg, Germany).

### 2.6. Determination of Pyocyanin

The determination of pyocyanin production was measured according to a previously described method, with a few minor modifications [15]. Briefly, bacteria in the log phase (10^8^ cfu/mL) were inoculated into LB broth supplemented with 5% LAB strain supernatant and incubated at 37 °C with shaking at 150 rpm for 24 h. After that, the culture (5 mL) was centrifuged (8000 rpm, 10 min) to obtain a supernatant from which pyocyanin was extracted with chloroform (3 mL). Then, 1 mL of 0.2 mol/L HCl was added to acidify the chloroform layer, and the absorbance value at 520 nm was measured.

### 2.7. Determination of Co-Aggregation of LAB and P. aeruginosa

The ability of LAB isolates to co-aggregate with *P. aeruginosa* was detected according to a previous method with slight modifications [16]. The culture medium of *P. aeruginosa* and LAB strains was centrifuged (4000 rpm, 10 min), washed thrice with PBS, and resuspended in 2 mL PBS to an OD_600_ of 0.50 ± 0.05 to standardize the number of bacteria (10^7^–10^8^ cfu/mL). Then, 2 mL aliquots of different *P. aeruginosa* and LAB strains were mixed and incubated at 37 °C for 2 h. Additionally, 4 mL aliquots of a single bacterial suspension (different LAB and *P. aeruginosa*) were incubated at 37 °C for 2 h as control tubes. After that, 1 mL of all the samples was measured at OD_600_. The determination of the co-aggregation rate was calculated using the following formula [16]:

co-aggregation rate (%) = [(A_x_ + A_y_) − 2 × A_t_]/(A_x_ + A_y_) × 100

where A_x_ and A_y_ denote the absorbance of LAB and *P. aeruginosa* at 0 h, respectively, while A_t_ denotes the absorbance of the mixture after 2 h.

### 2.8. Biological Properties of LAB

#### 2.8.1. Detection of AI-2 Signaling Molecules in LAB Supernatants

*V. harveyi* BB170 was used as an indicator bacterium for determining AI-2 by chemiluminescence. AI-2 can activate *V. harveyi* BB170 to produce fluorescence, and the fluorescence intensity was used to characterize the amount of AI-2. Autoinducer-2 (AI-2) was assayed based on the method described previously with slight modifications [17]. *V. harveyi* BB170 was incubated (2% inoculum) at 30 °C for 12 h until the cell OD_600_ was between 0.8 and 1.2 and then diluted proportionally (1:100) with fresh AB medium. The *V. harveyi* BB152 suspension and *V. harveyi* BB170 culture medium were used as the positive control and medium control, respectively. Diluted *V. harveyi* BB 170 was mixed with LAB supernatants and *V. harveyi* BB152 suspension at a volume ratio of 1:100 and incubated at 30 °C for 3.5 h. The chemiluminescence value was determined with a microplate reader. The intensity of the AI-2 signaling molecule was expressed as relative luminescence units (RLU). Relative fluorescence intensity (AI-2) of LAB = RLU_(LAB)_/RLU _(*V. harveyi* BB170)_.

#### 2.8.2. Determination of Biofilm Formation of LAB Strains

The biofilm formation assay of different LAB strains was carried out in a static environment in 96-disc polystyrene microplates with some modifications [18]. Briefly, *P. aeruginosa* was activated three times and grown in LB broth with shaking at 150 rpm at 37 °C for 12 h. An optical density (OD) of 0.05 was adjusted by LB broth at 600 nm. Then, *P. aeruginosa* cell suspension (0.05 OD) was incubated for 24 h at 37 °C in 96-disc microplates. After incubation, the plates were cleaned thrice with PBS before being stained for 10 min with 0.1% CV. After washing thrice with PBS, the plates were air-dried. The amount of biofilm formation was determined by adding 200 μL of 95% ethanol to bacteria-bound CV and measuring the absorbance at 600 nm with a microplate reader.

#### 2.8.3. Determination of the Auto-Aggregation of LAB Strains

The method to measure the auto-aggregation of LAB strains is referred to the protocol in Section 2.7. The LAB strains were activated three times and incubated for 18 h. The LAB strain was incubated for 18 h and washed with PBS, and adjusted to an OD_600_ of 0.50 ± 0.05. The LAB suspensions were then incubated for 1 h at 37 °C, and the supernatant’s absorbance was measured at 600 nm. The following formula was used to determine the auto-aggregation rate [16]:

auto-aggregation rate (%) = (A_0_ − A_t_)/A_0_ × 100

where A_0_ and A_t_ denote the absorbance values at 0 and 1 h, respectively.

### 2.9. Molecular Identification of LAB 

The LPyang were grown at 37 °C for 12 h, and the genomic DNA was extracted as per the manufacturer’s protocols of bacterial genome extraction kit (BIG, Guangzhou, China). The PCR primers of 16S rDNA were 16S rDNA-F (5′-AGA GTT TGA TCC ATG GCT CAG-3′) and 16S rDNA-R (5′AAG GAG GTG ATC CAG CC-3′). Then, PCR was performed using a previously described method [16]. The 16s rRNA gene was sent to BGI (Guangzhou, China) for sequencing. The sequences for the amplified 16S rDNA were searched using Blast in the NCBI databases to compare with the registered sequences.

### 2.10. Metabolomic Analysis of P. aeruginosa Antagonized by LPyang

*P. aeruginosa* was activated three times and grown in LB broth with shaking at 150 rpm at 37 °C for 12 h. *P. aeruginosa* overnight cultures (0.5 mL) were added to 2.7 mL of LB broth, MRS broth, or LPyang supernatant, respectively, and introduced to 50 mL of fresh LB broth and cultured at 37 °C for 24 h while being shaken at 150 rpm. The 15 mL sample was centrifuged at 4 °C (6000× *g*, 10 min), and the precipitated bacterial pellet was taken and washed twice in a 4 °C sterile 0.9% NaCl solution and then centrifuged at 4 °C (6000× *g*, 10 min). Bacterial pellets were immersed three times in liquid nitrogen, thawed on ice, and stored at −80 °C. For the metabolomic analysis, a 400 µL methanol/water (4:1, *v/v*) solution was added to a precisely weighed 50 mg solid sample and precipitated at −10 °C. A high-throughput tissue crusher Wonbio-96c (Shanghai Wanbo Biotechnology Co., Ltd., Shanghai, China) was used to process the combination for 6 min at 50 Hz, followed by 30 min of ultrasound at 40 kHz at 5 °C. The samples were left at −20 °C for 30 min and then centrifuged at 4 °C (13,000× *g*, 15 min), and the supernatant was subjected to LC-MS/MS analysis. L-2-chlorophenylalanine at 0.02 mg/mL was used as an internal standard. Chromatographic separation of the metabolites was performed on a Thermo UHPLC system equipped with an ACQUITY UPLC HSS T3 (100 mm × 2.1 mm i.d., 1.8 µm; Waters, Milford, MA, USA).

The metabolites’ characteristic mass spectra were found by searching trustworthy biochemical databases such as the Human Metabolome Database (HMDB) (http://www.hmdb.ca/, accessed on 20 April 2022) and the Metlin database (https://metlin.scripps.edu/, accessed on 20 April 2022) utilizing accurate mass, MS/MS fragment spectra, and isotope ratio differences. On the Majorbio Cloud Platform (https://cloud.majorbio.com, accessed on 20 April 2022), a multivariate statistical analysis was carried out using the ropls (Version 1.6.2, http://bioconductor.org/packages/release/bioc/html/ropls.html, accessed on 20 April 2022) R package from Bioconductor, including PCA, PLS-DA, and OPLS-DA. For the purpose of choosing prospective biomarkers, the VIP values and p values obtained from two-tailed *t*-tests (VIP > 1.0 and *p* < 0.05) were utilized as filtering criteria. On the Majorbio Cloud Platform (https://cloud.majorbio.com, accessed on 20 April 2022), metabolites with significant variations between the MRS negative control and the LPyang treatment were analyzed by pathway.

### 2.11. Data Processing

All the experiments were performed in two independent experiments with three replicates, and the results are expressed as the mean ± SD. Data analysis was performed using SPSS 28 (IBM, Armonk, NY, USA). Statistical analysis was performed to determine significant differences (*p* < 0.05) between the results using a Student’s *t*-test or ANOVA followed by Tukey’s post hoc test. GraphPad Prism 9 (GraphPad Software, Inc., La Jolla, CA, USA) was used to plot the various graphics.

## 3. Results

### 3.1. Effect of Antibacterial Activity of LAB on P. aeruginosa

The supernatants of 17 LAB strains could inhibit *P. aeruginosa* growth, while the antibacterial effect was different (Table 2). Among them, H5, H9, H11, H12, H14, and H17 showed a relatively strong antibacterial effect with an inhibition zone of about 20 mm, but the source and genus of the LAB were different, which indicated that the antibacterial ability of the LAB was strain-specific. The antibacterial effect of the LAB was significantly higher than that of the control (inhibition zone of acidified MRS (pH 4.0) was 9.12 ± 0.02.) (*p* < 0.05). 

### 3.2. Effect of LAB on the Biofilm Formation of P. aeruginosa

**Figure 1:**
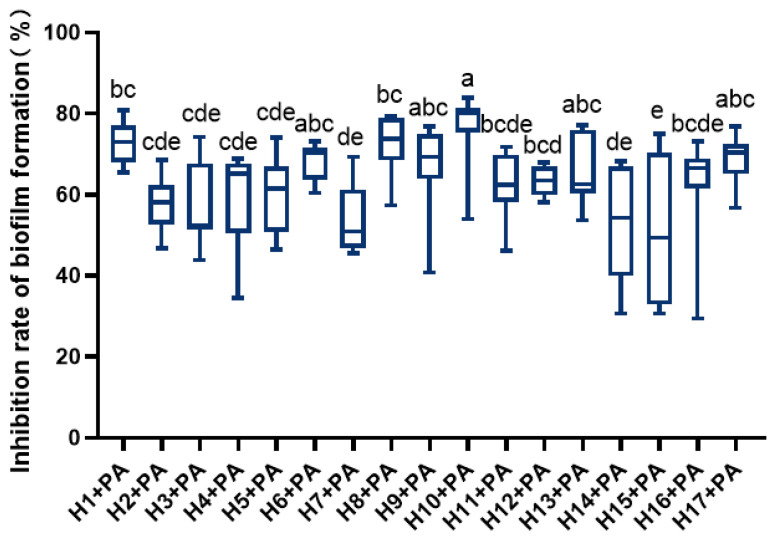
Inhibition rate of LAB against biofilm formation of *P. aeruginosa.* Differences are indicated by different superscript lowercase letters in the same column (*p* < 0.05). Statistical analysis was performed to determine significant differences (*p* < 0.05) between the results using Student’s *t*-test or ANOVA followed by Tukey’s post hoc test.

LAB significantly inhibited *P. aeruginosa*’s ability to form biofilms (Figure 1). Among them, H1, H6, H8, H10, and H17 showed higher inhibition of *P. aeruginosa* film formation, with H10 showing the strongest inhibition.

### 3.3. Effect of LAB on Pyocyanin Expression of P. aeruginosa

**Figure 2:**
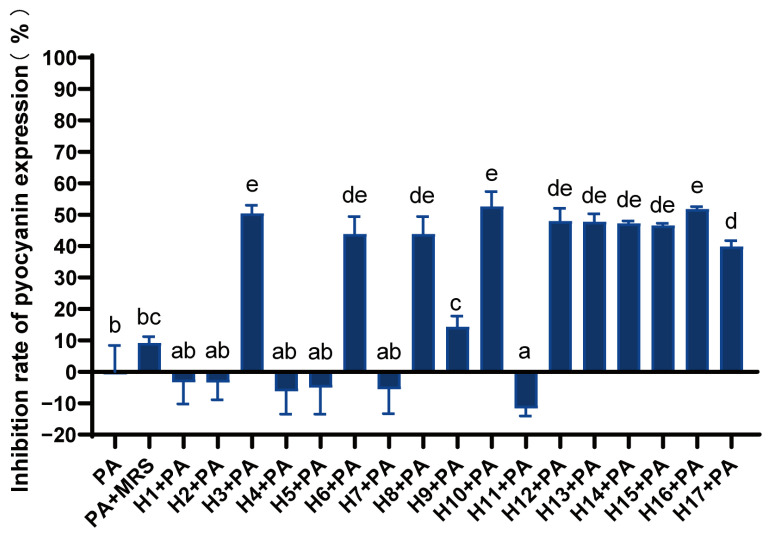
Inhibition rate of LAB against *P. aeruginosa* pyocyanin expression. Differences are indicated by different superscript lowercase letters in the same column (*p* < 0.05). Statistical analysis was performed to determine significant differences (*p* < 0.05) between the results using Student’s *t*-test or ANOVA followed by Tukey’s post hoc test.

Different LAB had different inhibitory effects on the production of pyocyanin (Figure 2). Strains H3, H6, H8, and H10–H17 could significantly inhibit pyocyanin expression, with the inhibition rate ranging from 40 to 50%. Additionally, strains H3, H10, and H16 had relatively high inhibition levels.

### 3.4. Analysis of Co-Aggregation Capacity of LAB and P. aeruginosa

**Figure 3:**
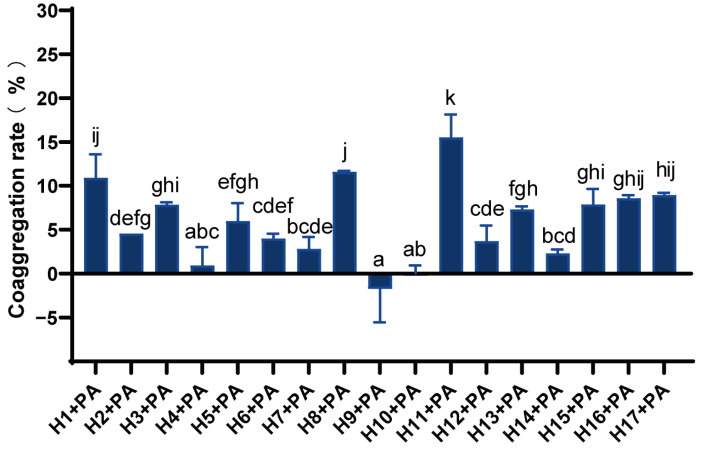
Co-aggregation rate of LAB and *P. aeruginosa*. Differences are indicated by different superscript lowercase letters in the same column (*p* < 0.05). Statistical analysis was performed to determine significant differences (*p* < 0.05) between the results using Student’s *t*-test or ANOVA followed by Tukey’s post hoc test.

There were significant differences in the co-aggregation rates of different LAB with *P. aeruginosa* (Figure 3). Strains H1, H8, and H11 had a strong co-aggregation capacity with co-aggregation rates above 10%, and in particular, strain H11 had the strongest co-aggregation activity of 15.52%. The coaggregation ability of strains H3, H5, H13, H15, H16, and H17 with *P. aeruginosa* was moderate, while that of H4, H9, and H10 was weak.

### 3.5. Biological Properties of LAB 

**Figure 4:**
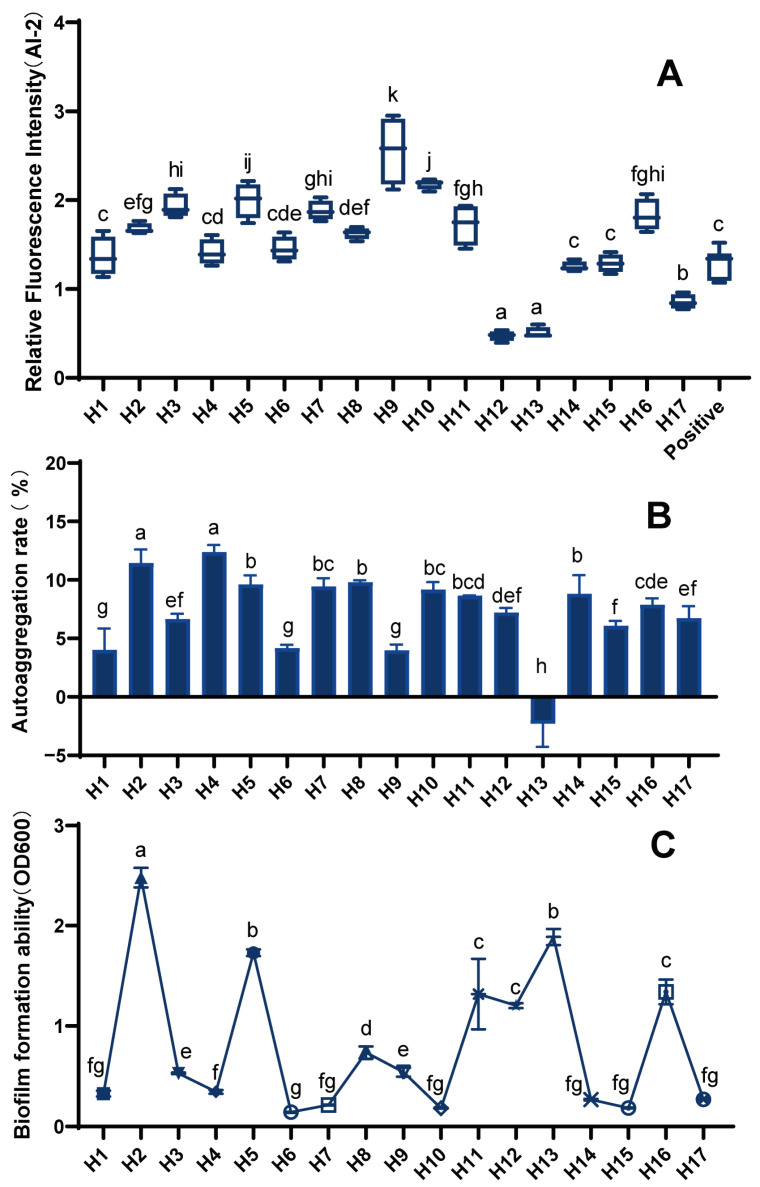
Biological properties of LAB. (**A**) AI-2 signaling molecule expression of LAB, (**B**) biofilm formation capacity of LAB, and (**C**) auto-aggregation rate of LAB) (differences are indicated by different superscript lowercase letters in the same column) (*p* < 0.05). Statistical analysis was performed to determine significant differences (*p* < 0.05) between the results using Student’s *t*-test or ANOVA followed by Tukey’s post hoc test.

In our study, we found that 10 LAB strains (H1, H2, H3, H5, H7–H11, and H16) had greater relative fluorescence intensity than the positive control and could produce strong AI-2 signaling molecules (Figure 4A). In contrast, strains H12, H13, and H17 produced no or only very weak AI-2 signaling molecules. 

The formation of biofilms can help beneficial microorganisms to resist the stress of unfavorable environments and resist antibiotics, thus achieving the effect of being engulfed by the immune system. Additionally, the aggregation of probiotics can also be carried out through biofilm formation, which greatly improves the activity and stability of strains; thus, research on biofilms is of great value in improving the probiotic properties of LAB. Different LAB strains differ significantly in biofilm formation (Figure 4B). Most of the 17 LAB strains could form biofilms, with strains H2, H5, H13, H11, H12, and H16 having a strong ability.

The functions of bacterial cell adhesion to biotic and abiotic surfaces are influenced by the bacteria’s auto-aggregation ability. Multiple receptor receivers on the surface of host tissues also have aggregation capacity [16]. Thus, the strong aggregation of bacteria is conducive to its adhesion to the host surface, which is beneficial for its probiotic functions. The auto-aggregation rate of LAB was significantly different between strains (Figure 4C). Eight strains, H2, H4, H5, H7, H8, H10, H11, and H14, all had a strong auto-aggregation capacity with rates of more than 8%.

### 3.6. Principal Component Analysis

**Figure 5:**
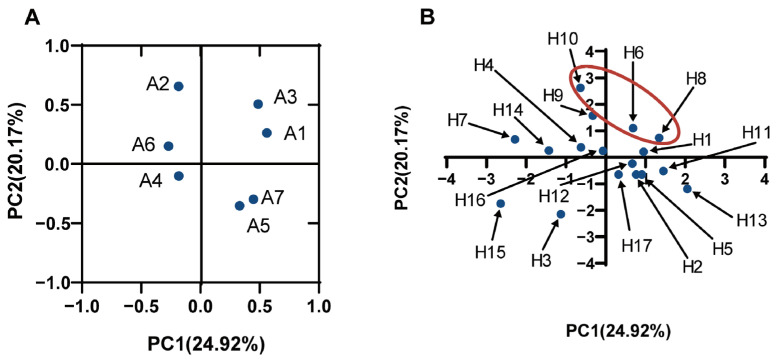
PCA analysis of various characteristics of LAB ((**A**) represents the factor load plot, A1 represents the inhibitory ability of LAB on *P. aeruginosa* growth; A2 represents the AI-2 fluorescence intensity of LAB supernatant; A3 represents the inhibitory ability of LAB on biofilm formation of *P. aeruginosa*; A4 represents the expression of chloropropria co-cultured with *P. aeruginosa*; A5 represents the coagulation capacity of LAB and *P. aeruginosa*; A6 represents the self-aggregation ability of LAB; A7 represents the ability of LAB to form biofilms; (**B**): represent the factor scoring plot, H1–H17 represents the LAB strains).

Principal component analysis (PCA) was performed to investigate the correlation among the various properties of LAB (auto-aggregation, co-aggregation, biofilm formation, and AI-2 expression of LAB) and the ability to inhibit *P. aeruginosa* growth, biofilm formation, and pyocyanin expression. The results, as seen in Figure 5A, were defined by four principal components (PC): (1) the first (PC1), which explained 24.92% of variables A1 (antibacterial), A3 (inhibitory ability against *P. aeruginosa* biofilm formation), and A7 (the biofilm formation ability of LAB); (2) the second (PC2) explained 20.17% of the variable A2 (the AI-2 of LAB); (3) the third (PC3) explained 18.55% of the variable A6 (the auto-aggregation ability of LAB); and (4) the fourth (PC4) explained 14.65% of the variables A4 (inhibition of pyocyanin expression) and A5 (co-aggregation ability). The first factor and the second factor were used to make a factor loading diagram, and, combined with the factor score diagram, it could be seen that the comprehensive evaluation scores of the three strains, H8, H6, and H10, were relatively high (Figure 5B). These three strains showed a relatively higher ability to inhibit the development of biofilms, growth, and pyocyanin expression of *P. aeruginosa* and have higher expression of AI-2; thus, these three strains were potential LAB that can antagonize *P. aeruginosa*. 

The 16S rRNA gene of H6 was amplified, purified, and sequenced by PCR, and H6 was identified as *Lactipantibacillus plantarum*, named *Lp. plantarum* LPyang. It was stored in the Wuhan Strain Preservation Center under the number CCTCC NO: M 2020158.

### 3.7. Effects of LPyang on P. aeruginosa Growth by Metabolome Analysis

**Figure 6:**
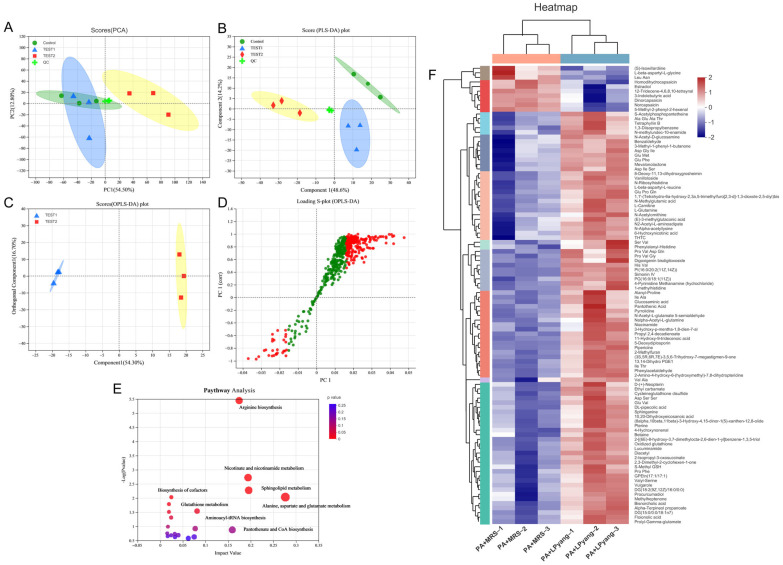
Metabolome analysis of *P. aeruginosa* culture with LPyang in ESI+ mode. (**A**) PCA, (**B**) PLS-DA, (**C**) OPLS-DA score plot; (**D**) S-loading plot based on OPLS-DA analysis; (**E**) heat map of liver metabolites with significant intergroup differences (VIP value > 1.0, *p* < 0.05) between MRS-negative control and LPyang-treated groups; (**F**) metabolic pathway prediction based on the KEGG database.

**Figure 7:**
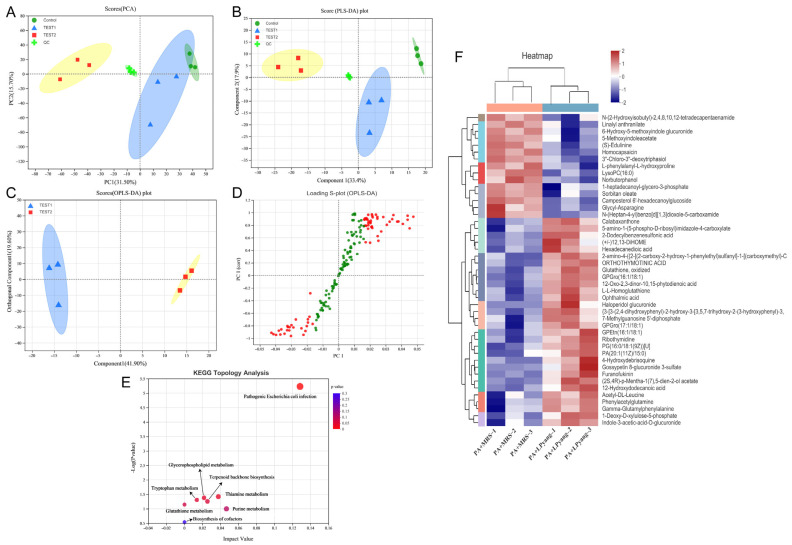
Metabolome analysis of *P. aeruginosa* culture with LPyang in ESI− mode. (**A**) PCA, (**B**) PLS-DA, (**C**) OPLS-DA score plot; (**D**) S-loading plot based on OPLS-DA analysis; (**E**) heat map of liver metabolites with significant intergroup differences (VIP value > 1.0, *p* < 0.05) between MRS-negative control and LPyang-treated groups; (**F**) metabolic pathway prediction based on the KEGG database.

In this study, the antagonistic mechanism of LPyang against *P. aeruginosa* growth was shown by UPLC-QTOF-MS, which was used to identify differences in the *P. aeruginosa* metabolome. Multivariate statistical analysis was used to compare the metabolomic data of *P. aeruginosa* samples and show the differences between experimental groups. The metabolic spectrum of the differences was separated in both ESI+ (Figure 6A,B) and ESI− (Figure 7A,B) ions modes, as shown by the score plots of PCA and PLS-DA, suggesting that the metabolite changes of *P. aeruginosa* induced by the LPyang supernatant were different compared to the MRS negative control. Furthermore, there was an obvious separation between the MRS and LPyang groups (Figure 6C and Figure 7C). The metabolomic profiles of *P. aeruginosa* differed between the MRS negative control and LPyang groups (Figure 6D and Figure 7D). Moreover, a criterion of VIP > 1.0 and *p* > 0.05 revealed a difference in 211 metabolites (ESI+ mode: 166 metabolites; ESI− mode: 45 metabolites) between the LPyang-treated and MRS-treated groups. The heat map indicated that there were 10 metabolites of *P. aeruginosa* (including (S)-isowillardiine, L-beta-aspartyl-L-glycine, Leu, Asn, homodihydrocapsaicin, estradiol, etc.) that decreased and 156 metabolites (including S-acetylphosphopantetheine, 1,3-diisopropylbenzene, N-methylundec-10-enamide, N-acetyl-D-glucosamine, pyrrolidine, N-acetyl-L-glutamate 5-semialdehyde, etc.) that increased in the ESI+ ion mode, compared to the MRS group. Additionally, 15 metabolites of *P. aeruginosa* (lysoPC, linalyl anthranilate, 6-hydroxy-5-methoxyindole glucuronide, 5-methoxyindoleacetate, (S)-edulinine, homocapsaicin, 3″-chloro-3″-deoxytriphasiol, etc.) were decreased, and 30 metabolites (calabaxanthone, 2-dodecylbenzenesulfonic acid, hexadecanedioic acid, etc.) were increased in the negative ion mode, compared to the MRS group (Figure 6E and Figure 7E).

The enrichment of different metabolite metabolic pathways of *P. aeruginosa* was used to assess the effects of LPyang intervention on the metabolic pathways of *P. aeruginosa* (Figure 6F and Figure 7F). The metabolic pathways affected by the LPyang intervention in the ESI+ and ESI− mode compared to the MRS-negative controls were glycerophospholipid metabolism, arginine biosynthesis, glutathione metabolism, alanine, aspartate, and glutamate metabolism, and aminoacyl-tRNA biosynthesis.

## 4. Discussion

*P. aeruginosa* is a conditional Gram-negative pathogen that may produce several extracellular virulence factors, leading to various diseases. Due to the application of antibiotics, many drug-resistant strains have appeared, making them more difficult to eradicate, and there are also strong side effects. The use of LAB against *P. aeruginosa* is considered a safe, natural approach because it is not toxic, has no sequelae, regulates intestinal flora, improves immune function, is antibacterial, etc. [19]. Zhang et al. [20] found that LAB have antibacterial activities against Gram-positive and Gram-negative bacteria. In addition, *Pediococcus pentosus* from fish and shrimp intestines had different inhibitory effects on *V. vulnificus, P. aeruginosa*, and *E. coli* [9]. LAB may exert antibacterial effects by producing bacteriocin and acidic substances such as organic acids. In this study, the Oxford cup bacteriostatic test showed that LAB could inhibit the growth of *P. aeruginosa*. Compared with the MRS medium at the same pH, the LAB cultures had a stronger ability to inhibit *P. aeruginosa* growth. It is evident that, in addition to the effect of acid, the metabolites of LAB can produce many types of inhibitory substances to antagonize *P. aeruginosa* growth, and different LAB could produce different inhibitory substances.

*P. aeruginosa* can easily form biofilms, which is the main reason for the difficulty in treating *P. aeruginosa* infections. The presence of biofilm prevents direct contact between bacteria and harmful substances and increases bacterial resistance to antimicrobials. However, many bacteria can attach to biofilms on the surface of different materials. Biofilm formation is thought to play a crucial role in improving survival from antibiotic exposure and can result in chronic infections. Therefore, the effectiveness of antibiotic therapy can be improved by inhibiting the biofilm formation of *P. aeruginosa*. *P. aeruginosa* biofilms consist of polysaccharides and extracellular DNA, essential for shielding bacterial colonies from external stress brought on by antimicrobial drugs [21]. Biofilm formation is frequently inhibited by biofilm dispersal [22,23], inhibition of quorum sensing [24], and iron chelators [25] because the development and dispersal of biofilms are controlled by a multifactorial process involving quorum-sensing systems, exopolysaccharides, and c-di-GMP. Furthermore, the impact of *P. aeruginosa* biofilm formation by LAB was studied for *Lp. plantarum* [26,27], *Lacticaseibacillus rhamnosus* [26], *Latilactobacillus curvatus* ZHG 2-1 [3], *L. lactis* [8], *L. fermentum* [28], etc. The main mechanism by which LAB inhibit *P. aeruginosa* biofilm formation is dependent on the low pH generated during LAB incubation. This is because metabolites of LAB could inhibit *P. aeruginosa* biofilm formation at an acidic pH but have no anti-biofilm activity at neutral pH [29]. In this study, 17 strains of LAB supernatants at pH 3.8 could inhibit *P. aeruginosa* biofilm formation, similar to the results of the above study. Therefore, LAB and their metabolites could be potential and valuable inhibitors of *P. aeruginosa*.

*P. aeruginosa* can produce a variety of phenazine compounds with redox activity, such as pyocyanin and phenazine-1-carboxylic acid. One of the most significant virulence factors secreted by *P. aeruginosa* is pyocyanin, which induces a variety of pathological alterations, including the degradation of the alveolar airspaces, goblet cell hyperplasia, and metaplasia [30]. Furthermore, different concentrations of pyocyanin can promote the growth of *P. aeruginosa*. Pyocyanin can increase infectivity and negative effects of *P. aeruginosa* on humans [31]. Moreover, pyocyanin also disrupts intracellular calcium homeostasis and inhibits the growth of epidermal cells, thus increasing the damage to the host [32]. Therefore, reducing the expression of pyocyanin is an effective approach for treating *P. aeruginosa* infections. In our study, LAB inhibited the expression of pyocyanin in *P. aeruginosa*, suggesting that LAB could reduce the intensity of infection.

LAB inhibited the growth, biofilm formation, and pyocyanin expression of *P. aeruginosa*. Moreover, some physiological characteristics of LAB promote their antibacterial ability. Co-aggregation is a process by which different bacteria adhere to each other through signal recognition molecules, which is closely related to the ability of pathogens to interact with each other. During coaggregation, probiotics come into close contact with pathogenic bacteria, and the antimicrobial substances produced by the former can directly and effectively inhibit pathogens by altering the microenvironment around them [16]. In the digestive tract, the coaggregation of LAB may create a barrier that supports the host defense mechanisms and hinders the entry and colonization of foodborne pathogens. LAB with probiotic potential are regarded as promising candidates for protective biofilm producers and could be identified by the presence of particular structural components surrounding their cytoplasmic membrane, including homopolysaccharides or heteropolysaccharides. LAB biofilms can form a natural barrier that inhibits pathogen invasion into the host, overcome peristalsis, and permanently colonize the gut epithelium, positively affecting human health [33,34]. AI-2, formed from the spontaneous rearrangement of 4, 5-dihydroxy-2, 3-pentanedione (DPD), is a universal quorum-sensing molecule that mediates intra- and interspecies communication between different bacteria and is the main QS molecule produced by most Gram-positive and Gram-negative bacteria [35]. AI-2 signaling molecules regulate many important physiological activities in bacteria, including toxin release, biofilm formation, and drug resistance [36]. LAB can produce AI-2, which regulates their physiological functions. Pathogen resistance and gut colonization are linked to the AI-2 activities of *Bifidobacterium breve* [37]. In *Bifidobacterium longum*, overexpression of *luxS* gene improves biofilm formation, which is used by probiotics for early colonization in the host [38]. Furthermore, in *Lp. plantarum*, the *Lp. plantarum* acid, antimicrobial activity, bile tolerance, and probiotic adhesion are all mediated by the AI-2/LuxS quorum-sensing system [39]. Therefore, in this study, the physiological characteristics of LAB in terms of coaggregation, biofilm formation, and AI-2 production were investigated, along with their ability to inhibit the growth, biofilm formation, and pyocyanin expression of *P. aeruginosa*, and the suitable strains were selected against *P. aeruginosa* by the PCA method. The results showed that strains H8, H6, and H10 had relatively high overall evaluation scores, and all three strains performed discs by the PCA method. H6, isolated from pickle, was selected as a LAB with potential inhibitory effects on *P. aeruginosa* for further study. Strain H6 was identified as *Lp. plantarum* LPyang by 16S rRNA.

To study the mechanism of LPyang against *P. aeruginosa*, metabolomic analysis of *P. aeruginosa* was explored. LPyang treatment significantly regulated the concentration of metabolites. Compared with the MRS negative control, PG(16:0/18:1(11Z)), PG(16:0/18:2(9Z,12Z)), PA(20:1(11Z)/15:0), and GPEtn(16:1/18:1), which are involved in glycerophospholipid metabolism, were strongly increased after LPyang treatment, except for lysophosphatidylcholine (LysoPC). The decrease in LysoPC levels in LPyang treatment may be due to the inhibition of phospholipid degradation, thus resulting in an accumulation of phospholipids [40].

LysoPC may regulate various cellular processes that affect the host, such as the regulation of monocyte adhesion molecules, chemoattractant properties, and secretion of pro-inflammatory cytokines by monocytes [41]. Therefore, LPyang may reduce the infectivity of *P. aeruginosa* to the host by inhibiting the expression of lysoPC. Glutathione is the key element in glutathione metabolism, a gamma-tripeptide composed of glutamic acid, cysteine, and glycine with an amide bond and sulfhydryl group, present in almost every cell of the body. It comes in two types: oxidized glutathione and reduced glutathione. Reduced glutathione has many physiological functions, such as antioxidation, anti-aging, immune enhancement, anti-tumor, etc. When reduced glutathione is transformed into oxidized glutathione, its function is weakened. In our study, oxidized glutathione increased in the LPyang treatment group, suggesting that LPyang can produce substances that turn reduced glutathione into oxidized glutathione, which increases the sensitivity of *P. aeruginosa* to the environment. In the metabolism of glycine, serine, and threonine, the LPyang-treated group had higher levels of betaine. As a secondary product of metabolism, betaine is a very important osmoregulator that enhances environmental stress tolerance. The increase in betaine compounds could improve the survival of *P. aeruginosa* in harsh environments [42]. This may be one of the reasons why LPyang treatment regulates the metabolism of *P. aeruginosa* rather than inhibiting its growth.

## 5. Conclusions

In this study, three LAB strains that can antagonize *P. aeruginosa* were selected by PCA analysis based on biological characteristics such as antagonizing *P. aeruginosa* activity, AI-2 expression, biofilm formation, co-aggregation ability, and pyocyanin expression. Among these LAB, *Lp. plantarum* LPyang showed a strong ability to inhibit the growth, biofilm formation, and pyocyanin expression of *P. aeruginosa*, which may be related to its biological characteristics of strong co-aggregation, biofilm formation, and AI-2-producing abilities. Metabolomic analysis showed that LPyang affects substances in the metabolic processes of *P. aeruginosa*, as indicated by changes in metabolites such as LysoPC, oxidized glutathione, and betaine, which may indicate that LPyang not only can inhibits the growth, biofilm formation, and pyocyanin expression of *P. aeruginosa*, but also affects its metabolic processes to reduce the infectivity of *P. aeruginosa*. Our study provides a research basis for finding substances of safe natural origin with antimicrobial properties, and LPyang will be further investigated to determine its primary antagonistic mechanism against *P. aeruginosa* in cellular and mouse models.

## Figures and Tables

**Table 1:** Information of strains.

Strain	Species	Origin	Strain	Species	Origin
H1	*Lactiplantibacillus plantarum (Lp. plantarum)*	Traditional koumiss	H10	*Ligilactobacillus salivarius*	Traditional koumiss
H2	*Lp. plantarum*	Traditional koumiss	H11	*Pediococcus pentosaceus*	Fermented bean curd
H3	*Lp. plantarum*	Traditional pickles	H12	*Ligilactobacillus salivarius*	Traditional pickles
H4	*Lactobacillus acidophilus*	ATCC4356	H13	*Pediococcus pentosaceus*	Fermented bean curd
H5	*Lactobacillus gallinarum*	Healthy poultry feces	H14	*Limosilactobacillus fermentum*	Healthy adult feces
H6	*Lp. plantarum*	Traditional pickles	H15	*Lp. plantarum*	Tibetan kefir
H7	*Lp. plantarum*	Traditional pickles	H16	*Lp. plantarum*	Fermented rice
H8	*Lactobacillus gasseri*	Traditional pickles	H17	*Lp. plantarum*	Traditional pickles
H9	*Ligilactobacillus salivarius*	Traditional koumiss			Traditional koumiss

**Table 2:** Antimicrobial activity of LAB against *P. aeruginosa*.

Strains	Inhibition Zone (mm)	Strains	Inhibition Zone (mm)
H1	17.27 ± 0.40 ^cde^	H10	18.13 ± 0.61 ^de^
H2	16.47 ± 0.83 ^de^	H11	20.13 ± 0.90 ^abc^
H3	15.47 ± 1.10 ^e^	H12	21.20 ± 2.92 ^ab^
H4	21.73 ± 0.11 ^a^	H13	18.00 ± 6.17 ^cde^
H5	20.13 ± 1.30 ^abc^	H14	19.53 ± 1.32 ^abc^
H6	18.27 ± 1.90 ^bcde^	H15	10.27 ± 3.57 ^f^
H7	12.33 ± 0.58 ^f^	H16	13.33 ± 0.64 ^e^
H8	22.27 ± 0.90 ^a^	H17	19.33 ± 0.39 ^abcd^
H9	19.27 ± 0.94 ^abcd^		

Notes: Differences are indicated by different superscript lowercase letters (*p* < 0.05). Statistical analysis was performed to determine significant differences (*p* < 0.05) between the results using Student’s *t*-test or ANOVA followed by Tukey’s post hoc test.

## Data Availability

The datasets generated for this study are available on request to the corresponding author.
